# Drugs and Medicines of North America

**Published:** 1887-12

**Authors:** 


### Drugs and Medicines of North America. A Quarterly. By J. U. & C. G. Lloyd. Cincinnati: J. U. & C. G. Lloyd, Publishers. 1887.

The fifth number of Volume II, dated June, 1887, is before us. We have repeatedly called the attention of our readers to this excellent journal. The number under review contains a fine description of Caulophyllum, illustrated with an unusually large plate, and a map showing its geographical distribution. As we have before shown in the description of other plants in this same journal, we have first its botany and natural history, then a description of the drug, next its commercial history, its pharmacopial history, with complete description of method of preparing it for use in medicine; its chemistry, its uses in the Eclectic School of Medicine, its uses in the Homœopathic School, and, finally, clinical illustrations.

To this number is appended a card from the editors, from which we quote the following:

"When this publication was commenced in 1884, the plan of issuing in parts was adopted as an experiment, partly to find out if such work was desired. It has been shown that the need of the work exists, and we are convinced beyond a doubt that our labors have been appreciated. It has been a financial success from the start, and we herein desire to again thank our numerous subscribers for the financial aid and encouragement in making the work a success. The main difficulty in our present plan is that of completing the subjects that must appear at stated times, and when we state that work commenced two years ago, without which the connected subjects would be incomplete, is not yet finished, we need add nothing farther. * * * * We have therefore decided to change the form of the work and to issue it hereafter in book form as a bound volume."

We wish the enterprise continued success in this new form, for the work is simply invaluable to all educated physicians. W. M. J.

